# Supplementary material for: Health Risk Assessment for Human Exposure to Heavy Metals via Food Consumption in Inhabitants of Middle Basin of the Atrato River in the Colombian Pacific
Source: Int J Environ Res Public Health. 2022 Dec 27;20(1):435. doi: 10.3390/ijerph20010435 (PMC9819723; doi:10.3390/ijerph20010435)
Supplement: Supplementary file 1 [file ijerph-20-00435-s001.zip › ijerph-1987960-supplementary.pdf]

Table S1. General characteristics of the population from the middle basin of the Atrato river (Chocó-Colombia) (n= 446).

Municipality	Weigth (kg)	Intake (days/week)		WCHA				GP			
		Fish	Vegetables	n (male)	n (female)	Age (years)	Range Age (years)	n (male)	n (female)	Age (years)	Range Age (years)
**Medio Atrato**	69.8 ± 16.0	3.2±0.6	4.0±1.4	11	74	30.99±11.5	15.19-49.78	74	49	50.20±17.3	18.01-88-36
**Bojayá**	72.6 ± 15.5	3.1±1.0	2.9±2.0	4	62	30.1±10.2	15.04-49.95	73	26	48.7±15.3	18.56-80.39
**Vigía del fuerte**	71.3 ± 13.2	3.3±0.6	3.2±2.3	10	83	30.57±10.7	15.36-48.21	89	46	62.34±9.9	18.25-82.05
**Murindó**	68.1 ± 14.4	4.0±1.1	3.3±1.9	5	72	31.8±11.1	15.19-49.09	64	34	52.8±17.1	18.70-88.81

GP: general population; WCHA: women of childbearing age.

Table S2. Concentrations of Hg, MeHg, As, Pb, and Cd (μg kg^{-1} ww) in fish of the Atrato region.

Scientific name(n)	Common name	Habitat	Hg	%MeHg	Pb	Cd	As	Fish length
**Medio Atrato**								
Hoplias malabaricus (17)	Quicharo	p	468.53±344.6	93.88±2.9	23.25±11.4	1.06±0.8	32.60±19.3	29.66±4.4
Trachelyopterus fisheri (5)	Caga	p	401.95±87.6	92.3±2.5	10.89±6.1	1.1±0.0	60.15±19.7	23.52±2.6
Pseudopimelodus schultzi (13)	Bagre	c	442.0±1096.8	95.2±1.8	13.6±7.1	1.1±0.5	17.2±8.0	51.6±6.8
Ctenolucius beani (5)	Agujeta	c	349.7±103.0	92.0±2.8	4.3±6.6	1.1±0.5	41.7±28.4	27.7±3.9
Caquetaia umbrifera (17)	Mojarra negra	c	123.4±56.3	90.3±3.1	16.0±6.2	1.1±0.9	46.12±18.1	22.4±3.6
Sternopygus aequilabiatus (6)	Beringo	c	168.2±632.4	88.6±3.0	6.7±15.1	1.1±2.7	60.1±9.1	38.9±13.2
Rhamdia quelen (21)	Barbudo	oc	165.8±235.2	93.5±3.2	16.6±9.4	1.1±2.3	18.3±25.2	21.4±2.7
Astyanax fasciatus (31)	Sardina	oc	212.8±133.9	92.5±3.5	4.3±3.7	1.1±0.5	46.9±25.9	10.6±3.5
Andinoacara pulcher (23)	Cocoa o Cocobolo	opv	144.5±54.7	87.2±3.1	17.6±8.2	1.1±0.6	14.3±9.0	11.5±2.2
Leporinus muyscorum (10)	Denton o liseta	o	109.1±32.4	92.7±2.7	14.1±28.6	1.1±0.9	41.3±24.3	31.0±2.4
Prochilodus magdalenae (12)	Bocachico	d	77.0±20.5	92.7±3.9	9.3±3.8	1.1±0.9	39.4±11.0	31.7±4.4
Hypostomus hondae (24)	Guacuco	d	140.5±43.9	81.4±4.8	22.5±10.5	1.1±0.6	134.2±61.0	21.2±5.1
Bojayá								
Hoplias malabaricus (11)	Quicharo	p	486.0±139.2	82.0±3.3	25.6±54.1	1.1±2.3	18.6±10.5	31.7±2.9
Ctenolucius beani (5)	Agujeta	c	1008.0±552.7	92.1±2.8	4.3±5.1	1.1±0.0	7.8±4.6	28.4±4.2

Prochilodus magdalenae (10)	Bocachico	d	136.3±43.6	88.1±6.2	17.8±11.0	1.1±2.3	16.0±22.7	25.7±1.7
**Vigía del Fuerte**								
Ageneiosus pardalis (6)	Doncella	P	990.9±364.6	96.3±2.3	15.3±5.6	1.1±0.4	42.6±17.1	47.0±18.2
Hoplias malabaricus (11)	Quicharo	p	486.6±193.6	77.1±2.7	4.3±11.8	1.1±2.6	11.9±95.6	33.0±3.5
Trachelyopterus fisheri (n=3)	Caga	p	754.2±331.6	93.4±4.8	4.3±7.3	1.0±0.0	2.7±1.6	24.2±3.2
Pseudopimelodus schultzi (6)	Bagre	p	560.5±222.7	75.9±3.5	16.4±1.0	1.1±2.4	12.1±71.1	48.0±7.7
Ctenolucius beani (4)	Agujeta	c	480.3±81.8	93.3±3.2	7.3±6.0	1.1±0.0	12.5±27.8	27.0±5.5
Sternopygus aequilabiatus (16)	Beringo	c	745.3±243.6	90.8±2.5	4.3±18.1	1.1±2.3	11.0±19.0	60.5±11.7
Cynopotamus atratoensis (2)	Boquiancha	c	729.6±105.7	89.5±7.9	4.3±0.0	1.1±0.0	12.5±2.1	14.5±3.5
Geophagus pellegrini (2)	Mojarra copetona	c	262.5±310.3	83.3±0.7	42.9±54.6	1.1±0.0	187.2±164.8	22.5±0.7
Caquetaia kraussii (10)	Mojarra amarilla o mojarra	oc	419.9±166.4	82.3±1.1	4.3±21.5	1.1±2.3	20.1±29.3	20.6±2.0
Rhamdia quelen (7)	Barbudo	oc	424.7±183.9	91.0±7.9	4.3±3.4	1.1±2.3	50.0±2.0	25.9±4.4
Pimelodus sp (6)	Gunguma	oc	147.9±277.4	96.3±1.6	9.0±41.5	1.1±2.4	21.9±16.9	27.7±12.1
Andinoacara pulcher (1)	Cocoa o Cocobolo	opv	41.48	99.21	11.10	LDM	18.94	11.0
Leporinus muyscorum (1)	denton o liseta	o	96.64	87.33	40.26	LDM	58.17	27.5
Prochilodus magdalenae (22)	Bocachico	d	95.3±36.9	91.4±4.4	4.33±27.9	1.1±0.5	30.3±14.7	25.0±3.8
Hypostomus hondae (10)	Guacuco	d	41.5±32.5	88.0±16.3	4.33±4.7	1.1±0.4	33.1±27.6	20.9±6.2
**Murindó**								
Ageneiosus pardalis (22)	Doncella	p	700.9±202.2	88.0±7.6	17.1±22.4	1.1±0.2	26.8±48.6	27.0±5.2
Hoplias malabaricus (10)	Quicharo	p	668.8±89.5	75.6±6.0	12.5±8.5	1.1±0.4	21.9±26.0	35.8±2.3
Trachelyopterus fisheri (6)	Caga	p	606.9±224.6	95.8±1.7	10.2±7.8	1.1±2.4	4.5±2.0	22.1±3.6
Pseudopimelodus schultzi (1)	Bagre	p	348.88	71.08	LDM	LDM	5.91	19
Ctenolucius beani (1)	Agujeta	c	961.51	96.06	LDM	LDM	19.03	30
Sternopygus aequilabiatus (1)	Beringo	c	657.13	71.98	15.362	LDM	LDM	67
Caquetaia kraussii (10)	Mojarra amarilla o mojarra	oc	511.8±231., 5	94.4±2.4	22.3±14.3	1.1±2.3	10.0±8.9	20.7±3.3
Rhamdia quelen (16)	Barbudo	oc	542.6±186.6	85.3±9.6	18.5±29.5	1.1±0.3	20.6±208.9	27.0±1.1
Astyanax fasciatus (7)	Sardina	oc	84.8±116.2	92.9±2.6	12.1±155.6	1.1±0.7	13.9±7.9	8.4±2.0
Pimelodus punctatus (2)	Charre	oc	193.5±43.5	73.8±1.7	4.3±0	1.1±0.0	8.0±7.4	21.5±2.1
Pimelodella chagresi (10)	Micudo	oc	146.9±82.5	89.6±7.6	4.3±7.1	1.06±2.3	6.6±3.2	11.9±2.3
Andinoacara pulcher (2)	Cocoa o Cocobolo	opv	32.9±4.3	84.8±3.4	136.6±187.1	1.1±0.0	7.8±7.1	7.85±8.7
Leporinus muyscorum (3)	Dentón o Liseta	o	44.5±22.7	87.0±5.2	103.9±1.0	1.1±0.0	28.8±26.3	25.4±1.4
Prochilodus magdalenae (26)	Bocachico	d	134.4±45.9	91.6±4.1	21.3±34.7	1.1±0.4	24.5±26.5	24.5±2.8

Hypostomus hondae (6)	Guacuco	d	45.6±15.9	86.2±3.2	12.0±7.3	1.1±2.4	86.3±56.1	24.5±3.6

* Habits: P: piscivore, C: carnivore, OC: omnivore with a carnivorous tendency, OPV: omnivore with a preference for fish and plant material, O: omnivore, D: detritivore; (Nd) concentrations not determined, the concentrations in (μg kg^{-1}) are established with the median, the mean and the respective standard deviation for each species of fish in the middle basin of the Atrato River. LDM is the detection limit of the concentrations of each metal (Pb: 4.33, As: 2.71, Cd: 1.06). The order of the municipalities is from upstream to downstream in the Atrato River.

Table S3. Concentrations of heavy metals (mg/kg) and consumption (days/week) of fruits and vegetables in the middle basin of the Atrato river.

Vegetable species	Common Name	Consumption (days/week)	Food consumption (g/kg)	Heavy metals concentrations			
				Hg	Pb	Cd	As
**Medio Atrato**	**Fruits – tubers (14)**						
Colocasia esculenta	Achin	2.6±1.4	572±10	2.62±0.3	28.34±0.9	LDM	9.51±1.5
Oryza sativa	Rice	6.3±1.6	125±5	12.02±2.2	LDM	LDM	28.75±5.3
Alibertia patinoi	Borojó	4.0±1.7	122.5±6	3.97±1.8	LDM	LDM	203.16±10.3
Musa × paradisiaca	Plantain	5.5±2.1	400±20	2.90±0.5	LDM	LDM	3.18±0.3
Musa balbisiana	Banana	4.5±2.1	640±30	1.34±0.1	LDM	9.88±2.3	LDM
Cocos nucifera	Coconut	4.2±2.6	645±25	12.39±3.3	LDM	2.89±0.3	LDM
Citrus aurantifolia	Lemon	5.8±1.8	135±5	0.39±0.03	Nd	Nd	Nd
Musa sp	Papoche	4.8±2.5	610±30	3.12±0.3	10.63	LDM	12.49±1.3
Musa sapientum	Primitivo	3.1±1.1	436±15	3.14±0.2	LDM	LDM	9.96±0.8
Solanum sessilliflorum Dunal	Lulo	4.0±0.0	300±13	3.27±0.5	LDM	8.10	LDM
Zea mays	Corn	4.3±1.5	200±18	3.91±0.9	LDM	LDM	38.16±2.3
Dioscorea trifida	Yam	2.4±0.8	495±32	2.88±0.3	LDM	LDM	LDM
Carica papaya	Papaya	.6±1.1	300±22	4.16±0.4	8.13±0.8	3.36±0.3	7.97±0.5
Manihot esculenta	Yucca	3.3±1.7	420±20	2.06±0.1	LDM	LDM	LDM
	**Vegetables- Stem(4)**						
Ocimum campechianum	Basil	3.0±1.3	140±10	23.42±5.3	63.10±10.3	11.01±2.3	110.41±20.3
Eryngium foetidum	Cilantro	5.6±1.4	50±5	21.11±4.3	39.11±6.3	9.98±3.3	76.12±20.3
Origanum vulgare	Oregano	0.9±1.0	30±2	42.94	66.71	25.34	146.72±25.3
Saccharum officinalis	Cane	4.3±2.5	462±16	2.38±0.4	Nd	Nd	Nd
	Total(18)	4.0±1.4		8.2±10.9	16.2±21.5	5.3±6.5	41.2±61.2
**Bojayá**	**Fruits – tubers (9)**						
Colocasia esculenta	Achin	2.4±0.9	572±10	1.87±0.1	LDM	50.92±10.3	29.38±9.3
Musa balbisiana	Banana	3.3±1.8	640±15	1.55±0.5	LDM	4.26±0.9	LDM
Manihot esculenta	Yucca	2.8±1.5	420±9	2.40±0.8	LDM	2.17±0.3	13.07±3.6
Musa × paradisiaca	Plantain	5.8±2.1	400±35	2.06±0.5	LDM	3.46±1.3	18.11±4.3
Citrus aurantifolia	Lemon	7.0±0.0	135±10	1.18±0.3	LDM	462.69	50.06±9.3
Musa sp	Papoche	3.7±1.5	610±26	2.71±0.6	LDM	LDM	LDM
Alibertia patinoi	Borojo	1.5±0.9	122.5±10	1.80±0.1	LDM	LDM	6.73±7.3
Solanum sessilliflorum Dunal	Lulo	1.2±1.0	300±29	2.65±0.3	LDM	8.22±2.3	LDM
Zingiber officinale	Ginger	1.3±1.0	33±2	9.01±2.3	LDM	12.63	50.54±7.3
	**Vegetables- Stem (7)**						

Ocimum campechianum	Basil	4.3±1.2	140±10±20.2	9.99±1.9	LDM	11.39±2.3	391.75±30.8
Eryngium foetidum	Cilantro	5.2±1.7	50±3	16.3±8.3	LDM	57.84	74.36±9.3
Origanum vulgare	Oregano	1.6±1.0	30±1	10.75±4.3	LDM	32.99	70.00±4.3
Minthostachys mollis	Poleo	1.2±1.0	50±4	10.48±7.3	Nd	Nd	Nd
Basella rubra var	Spinach	0.3±0.6	60±10	15.31±4.3	LDM	14.78	27.33±9.3
Allium fistulosum L	Chives	3.6±1.3	0.85±0.4	6.73±2.3	9.99	23.06	60.54±10.6
Saccharum officinalis	Cane	0.03±0.01	462±35	2.34±0.7	Nd	Nd	Nd
	Total(16)	2.9±2.0		6.1±5.1	4.7±1.5	49.0±120.4	57.1±99.6
**Vigía del fuerte**	**Fruits – tubers (9)**						
Colocasia esculenta	Achin	2.4±0.9	572±18	2.46±0.3	Nd	Nd	Nd
Oryza sativa	Rice	6.7±1.3	125±10	1.51±0.1	LDM	LDM	32.54±10.0
Citrus aurantifolia	Lemon	4.3±2.5	135±8	0.29±0.2	Nd	Nd	Nd
Musa balbisiana	Banana	3.9±2.5	640±30	1.68±0.4	LDM	LDM	LDM
Zea mays	Corn	2.7±1.5	200±20	1.95±0.3	LDM	LDM	26.75±9.5
Musa sp	Papoche	3.6±2.8	610±35	1.70±0.2	LDM	LDM	12.55±2.5
Musa × paradisiaca	Plantain	6.3±1.9	400±23	2.19±0.3	LDM	LDM	38.45±8.9
Manihot esculenta	Yucca	2.7±1.5	420±16	3.30±0.5	LDM	5.65±2.3	28.95±9.3
Solanum sessilliflorum Dunal	Lulo	4.5±3.5	300±19	1.87±0.2	7.07±1.3	4.82±1.3	16.66±7.9
	**Vegetables- Stem (7)**						
Ocimum campechianum	Basil	5.8±1.2	140±10	15.02±4.9	LDM	12.65±1.3	101.54±20.8
Eryngium foetidum	Cilantro	6.4±0.5	50±3	9.58±2.3	LDM	6.73	75.35±10.3
Origanum vulgare	Oregano	0.03±0.01	30±2	17.35±3.3	15.32	12.45	121.60±10.9
Ocimum basilicum L	White basil	2.7±1.1	140±6	7.85	21.50	LDM	89.54±10.9
Minthostachys mollis	Poleo	0.04±0.01	50±2	9.85	Nd	Nd	Nd
Basella rubra var	Spinach	0.04±0.02	60±9	11.05	4.33	LDM	14.75±4.3
Saccharum officinalis	Cane	0.05±0.01	462±25	2.20±0.8	Nd	Nd	Nd
	Total(16)	3.2±2.3		5.6±5.4	6.9±5.6	4.1±4.4	46.8±39.6
**Murindó**	**Fruits – tubers (8)**						
Colocasia esculenta	Achin	2.4±0.9	572±17	3.82±0.9	LDM	16.54±0.9	29.93±5.3
Musa balbisiana	Banana	4.6±2.2	640±10	7.33±1.3	LDM	LDM	6.89±1.3
Musa sapientum	Primitivo	5.6±2.0	436±10	4.54±1.8	LDM	LDM	9.78±0.9
Musa × paradisiaca	Plantain	6.5±1.3	400±25	2.84±0.3	LDM	1.94±0.3	39.41±9.3
Manihot esculenta	Yucca	2.8±1.6	420±22	3.99±0.9	LDM	35.79±12.3	19.84±4.3
Citrus aurantifolia	Lemon	4.7±1.0	135±10	0.30±0.3	Nd	Nd	Nd
Alibertia patinoi	Borojo	2.8±1.0	122.5±11	2.04±0.8	LDM	3.46±0.9	6.61±1.2
Zea mays	Corn	0.04±0.01	200±10	2.38±0.9	LDM	1.62±0.3	26.43±5.3
	**Vegetables- Stem (6)**						
Eryngium foetidum	Cilantro	4.1±1.5	50±6	9.87±0.8	LDM	2.80±0.4	77.94±10.5
Ocimum basilicum L	White basil	3.8±1.2	140±14	6.96±0.3	LDM	Nd	Nd
Minthostachys mollis	Poleo	0.9±1.0	50±10	19.26±3.3	LDM	LDM	352.82±10.9
Ocimum tenuiflorum	Purple basil	4.0±1.0	140±15	10.03±1.3	LDM	LDM	Nd
Allium fistulosum L	Chives	4.1±1.2	0.85±0.6	6.67±0.3	LDM	LDM	38.72±9.3
Saccharum officinalis	Cane	0.03±0.01	462±18	1.32±0.4	Nd	Nd	Nd
	Total (14)	3.3±1.9		5.8±4.9	4.3±0.0	6.1±10.8	60.84±107.8

The quantifications of the mean concentrations were carried out using the average and the standard deviation is taken for the plants for each municipality of the middle basin of the Atrato river,

established for the not obtained concentrations. Nd: concentrations not determined; LDM: is the detection limit of the concentrations of each metal.

Table S4. Estimation of the potential risk to the population from fish consumption in the Atrato river basin.

Fish species	THg	MeHg	%MeHg	FIR	IR	GP			WCHA			Pi[b]	Pi[c]
						**EWI**	MeHg permissible	MFW	EWI	MeHg permissible	MFW		
**Medio Atrato**													
Hoplias malabaricus	468.54	**432.99**	93.50	3.0	**768**	**4.38**	283.54	502.92	**4.74**	291.95	**517.85**	0.86	**2.16**
Trachelyopterus fisheri	412.38	**395.16**	91.29	2.3	**588.8**	**3.33**	239.07	564.83	**3.32**	380.81	**567.62**	0.79	1.97
Pseudopimelodus schultzi	442.02	**423.65**	95.14	3.0	**768**	**4.38**	283.54	514.01	**4.66**	291.95	**529.27**	0.84	**2.11**
Ctenolucius beani	373.50	**341.20**	91.39	Nd	**998.4**	**5.0**	218.10	638.22	**4.86**	224.58	**657.17**	0.68	1.70
Caquetaia umbrifera	109.44	99.46	90.88	Nd	588.8	0.86	369.83	2189.22	0.83	380.81	2254.43	0.19	0.49
Sternopygus aequilabiatus	168.24	148.43	88.23	2.6	665.6	1.45	327.16	1467.08	1.40	336.87	1510.63	0.29	0.74
Rhamdia quelen	165.76	143.46	93.92	3.2	819.2	1.72	265.82	1517.12	**1.67**	273.71	1562.99	0.28	0.71
Astyanax fasciatus	212.82	188.68	92.47	3.6	921.6	2.55	236.28	1154.12	**2.48**	243.29	1188.38	0.37	0.94
Andinoacara pulcher	142.26	117.79	87.47	3.0	768	1.32	283.54	1848.72	1.29	291.95	1903.60	0.23	0.58
Leporinus muyscorum	117.56	106.53	92.69	3.3	844.8	1.32	257.76	2044.17	1.28	265.41	2104.85	0.21	0.53
Prochilodus magdalenae	79.67	72.86	93.66	4.3	1100.8	1.17	197.81	2988.65	1.14	203.69	3077.37	0,14	0.36
Hypostomus hondae	132.85	101.89	82.30	4.3	1100.8	1.32	197.81	2137.21	1.60	203.69	2200.65	0.20	0.50
**Bojayá**													
Hoplias malabaricus	447.63	**358.96**	81.69	3.1	**793.6**	**3.93**	291.73	644.98	**4.05**	**283.46**	**626.70**	071	1.79
Ctenolucius beani	1007.98	**963.72**	92.28	2.0	**512**	**6.81**	452.18	240.23	**7.01**	**439.37**	**233.42**	1.92	**4.81**
Prochilodus magdalenae	142.04	126.37	87.98	4.1	1049.6	1.83	220.57	1832.05	**4.88**	214.32	1780.14	0.25	0.63
**Vigía del fuerte**													
Ageneiosus pardalis	1006.26	**956.82**	97.01	3.1	**793.6**	**15.03**	203.19	233.77	**14.51**	**210.83**	**242.67**	1.91	**4.78**
Hoplias malabaricus	471.62	**374.22**	75.77	3.3	**844.8**	**4.52**	264.77	597.71	**4.75**	**274.84**	**620.45**	0.75	1.87
Trachelyopterus fisheri	842.86	**794.90**	94.30	2.7	**691.2**	**7.86**	323.61	281.39	**7.57**	**335.92**	**292.10**	1.58	**3.97**
Pseudopimelodus schultzi	586.04	**459.35**	75.34	3.5	**896**	**5.88**	249.64	486.93	**6.57**	**259.14**	**505.48**	0.92	**2.30**
Ctenolucius beani	460.84	**439.84**	94.19	3.0	**768**	**4.83**	291.25	508.54	**4.65**	**302.33**	**527.89**	0.88	**2.20**
Sternopygus aequilabiatus	772.82	**681.65**	90.30	3.1	**793.6**	**7.73**	281.85	328.14	**7.45**	**292..58**	**340.63**	1.36	**3.40**

Cynopotamus atratoensis	729.60	**648.91**	89.51	2.0	**512**	**4.75**	436.87	344.69	**4.57**	**453.50**	**357.81**	1.30	**3.24**
Geophagus pellegrini	262.54	**219.72**	83.26	4.0	1024	**3.21**	218.43	1018.02	**3.10**	226.75	1056.76	0.44	1.09
Caquetaia kraussii	402.53	**335.63**	82.63	3.2	**819.2**	**3.93**	273.04	666.44	**3.78**	**283.43**	**691.80**	0.67	1.68
Rhamdia quelen	456.27	**456.80**	95.58	2.7	**691.2**	**4.51**	323.61	489.66	**4.35**	**335.92**	**508.30**	0.91	**2.28**
Pimelodus sp	147.92	143.87	95.99	Nd	691.2	1.42	323.61	1554,69	1.37	335.92	1613.86	0.29	0.71
Andinoacara pulcher	41.48	41.16	99.21	3.6	921.6	0.74	242.70	5434.69	0.52	291.94	5641.26	0.08	0.21
Leporinus muyscorum	96.64	84.40	87.33	4.0	1024	1.23	218.43	2650.19	1.19	226.75	2751.04	0.16	0.42
Prochilodus magdalenae	96.76	82.96	91.80	4.0	1024	1.21	218.43	2696.18	1.17	226.75	2798.79	0.16	0.41
Hypostomus hondae	29.91	25.73	87.97	3.3	844.8	0.31	264.77	8693.91	0.29	274.84	9024.75	0.05	0.12
**Murindó**													
Ageneiosus pardalis	700.89	**589.08**	88.60	4.3	**1100.8**	**9.70**	194.27	363.03	**9.53**	197.61	369.18	1.17	**2.94**
Hoplias malabaricus	689.33	**485.71**	77.94	4.4	**1126.4**	**8.18**	189.85	440.29	**8.04**	193.12	447.86	0.97	**2.42**
Trachelyopterus fisheri	711.74	**673.38**	96.05	2.0	**512**	**5.15**	417.68	317.58	**5.07**	424.87	523.05	1.34	**3.36**
Pseudopimelodus schultzi	348.88	**248.00**	71.08	6.2	**1587.2**	**5.89**	134.73	862.28	**5.79**	137.05	877.12	0.49	1.24
Ctenolucius beani	961.52	**923.64**	96.06	2.5	**640**	**8.84**	334.15	231.53	**8.69**	339.9	235.51	1.84	**4.61**
Sternopygus aequilabiatus	657.14	**473.05**	71.98	3.9	**998.4**	**7.06**	214.19	252.07	**6.94**	217.88	459.85	0.94	**2.36**
Caquetaia kraussii	506.85	**471.97**	93.84	3.6	**921.6**	**6.50**	232,04	453.10	**6.39**	236.04	460.90	0.94	**2.35**
Rhamdia quelen	524.42	**458.17**	84.08	4.9	**1254.4**	**8.79**	170.48	466.76	**8.45**	170.41	474.79	0.91	**2.29**
Astyanax fasciatus	84.78	78.04	93.46	4.1	1049.6	1.22	203.75	2740.48	1.20	207.25	2787.64	0.16	0.39
Pimelodus punctatus	193.48	142.42	73.80	2.5	640	1.36	334.15	1501.54	1.34	339.9	1527.38	0.28	0.71
Pimelodella chagresi	118.04	131.69	89.57	3.2	819.2	1.61	261.05	1623.92	1.58	265.54	1651.87	0.26	0.65
Andinoacara pulcher	32.9	27.97	84.83	3.5	896	0.37	238.67	7643.47	0.36	242.78	7775.00	0.05	0.13
Leporinus muyscorum	32.69	27.69	84.73	5.6	1433.6	0.59	149.17	7720.72	0.58	171.54	7853.58	0.05	0.13
Prochilodus magdalenae	124.69	113.50	91.29	4.8	1228.8	**2.08**	174.03	1884.15	**2.05**	177.03	1916.58	0.22	0.56
Hypostomus hondae	44.17	38.86	85.96	3.7	947.2	0.55	225.77	5502.29	0.54	229.66	5596.97	0.07	0.19

THg and MeHg (μg/kg) are mercury and methylmercury concentrations, FIR (g/day) is the food ingestion rate, IR (g/week) is the weekly intake of fish, EWI (μg/kg bw/week) is the Estimated Weekly Intake of MeHg, MeHg permissible is the permissible safety level (μg/kg), and MFW (g/week) is the estimated maximum amount of fish that can be weekly consumed per person. Pi: pollution index. The order of the municipalities is from upstream to downstream on the Atrato River. [a]For calculation the PTWI 3.2 μg/kg bw/week for the adult population and 1.6 μg/kg bw/week for vulnerable population were used. [b]For Pi calculation the threshold value of 500 μg/kg ww for adult populations was used. [C]For Pi calculation the

threshold value of 200 μg/kg ww for vulnerable populations was used. The highlighted bold values are above those established in the case of MeHg concentrations, the weekly IR values that are above the MFW, and the Pi values that are in Pollution Degree 2, 3, and 4.

Table S5. Classification of pollution index of metals (Zhang et al., 2019)

**Grade**	**Individual factor contamination index criteria**	
	**Pollution index**	**Pollution degree**
Grade 1	Pi ≤ 1	Clean
Grade 2	1 < Pi ≤ 2	Slight pollution
Grade 3	2 < Pi ≤ 3	Moderate pollution
Grade 4	Pi > 3	Strong pollution
